# Effects of chest physiotherapy and aerobic exercise training on physical fitness in young children with cystic fibrosis

**DOI:** 10.1186/1824-7288-38-2

**Published:** 2012-01-10

**Authors:** Bulent Elbasan, Nur Tunali, Irem Duzgun, Ugur Ozcelik

**Affiliations:** 1 Department of Physiotherapy and Rehabilitation, Faculty of Health Sciences, Gazi University, Muammer Yasar Bostanci, Ankara, postcode 06500, Turkey; 2 Department of Physiotherapy and Rehabilitation, Faculty of Health Sciences, Medipol University, Ataturk Bulvari, Istanbul, postcode 34083, Turkey; 3 Department of Child Pulmonary Diseases, Faculty of Medicine, Hacettepe University, Samanpazari, Ankara, postcode 06100, Turkey

**Keywords:** Cystic fibrosis, physical fitness, aerobic exercise, training

## Abstract

**Background:**

Cystic fibrosis is a multisystem disease where the main problems are existing in the respiratory system. Aerobic exercise programs are effective in increasing physical fitness and muscle endurance in addition to chest physiotherapy.

**Objective:**

The aim of this study was to evaluate the effects of chest physiotherapy and aerobic exercise training on physical fitness in young children with cystic fibrosis.

**Methods:**

Sixteen patients with cystic fibrosis, between the ages 5-13 years, were included in this study. All children were assessed at the beginning and at the end of 6 week of the training. Modified Bruce protocol was used for assessing the cardiovascular endurance. The sit-up test was used to evaluate the dynamic endurance of abdominal muscles, standing long jump was used to test power, sit and reach, trunk lateral flexion, trunk hyperextension, trunk rotation and forward bending tests were used to assess flexibility, 20 m shuttle run test and 10-step stair climbing tests were used to assess power and agility. All patients received chest physiotherapy and aerobic training, three days a week for six weeks. Active cycle of breathing technique and aerobic exercise training program on a treadmill were applied.

**Results:**

By evaluating the results of the training, positive progressions in all parameters except 20 m shuttle run and 10 stairs climbing tests were observed (p < 0.05). Active cycle of breathing techniques were used together with exercise training in clinically stable cystic fibrosis patients increases thoracic mobility (p < 0.05) and the physical fitness parameters such as muscle endurance, strength and speed (p < 0.05). Comparison of the results in sit and reach and forward bending tests were not significant (p > 0.05).

**Conclusion:**

It is thought that in addition to medical approaches to the systems affected, the active cycle of breathing techniques along with aerobic training helps to enhance the aerobic performance, thoracic mobility and improves physical fitness in children with cystic fibrosis.

## Background

Cystic fibrosis is a multisystem disease where the main problems are existing in the respiratory system [1]. Most common problems requiring help in these patients are excessive bronchial secretion, decreased exercise tolerance and feeling of breathlessness [2,3]. Aerobic exercise programs are effective in increasing physical fitness and muscle endurance in addition to chest physiotherapy [4]. It is therefore important to provide pulmonary rehabilitation programs in addition to regular medical treatment to increase the quality of life and prolong survival in children with cystic fibrosis.

Several studies have demonstrated favorable effects of exercise programs in children and adults with cystic fibrosis [5,6]. Specifically, an increase in physical activity was associated with an improvement in exercise capacity, stabilization and improvement in lung function and a higher quality of life [5,6].

There are some studies which support that peripheral muscle strength, as a physical fitness parameter, can be affected in children with cystic fibrosis [7,8]. Aerobic exercise training in addition to chest physiotherapy is a vital component of the rehabilitation program of cystic fibrosis patients. Exercise is known to increase cardiorespiratory fitness and muscle endurance, decrease the feeling of shortness of breath and support a feeling of wellness [2,9]. It was reported that aerobic exercise increases forced expiration and sputum clearance in children with cystic fibrosis. This has led to an improvement in the clinical state, exercise tolerance, cardiovascular fitness and muscular endurance, together with a decreased sensation of breathlessness [10-12].

Cystic fibrosis patients who were included in an aerobic exercise program (walking and running) three days a week for three weeks at 70% of the maximum heart rate showed increase in respiratory muscle endurance and improvement in aerobic performance [12].

In the literature, the data showing the changes created by active cycle of breathing techniques and aerobic exercise training in children with cystic fibrosis on physical fitness parameters are inadequate and generally from the adult patients with cystic fibrosis. The aim of this study was to evaluate the effects of active cycle of breathing techniques and aerobic exercise training on physical fitness in young children with cystic fibrosis.

### Patients and Methods

A total of 20 clinically stable children between the ages 5-13 were included in the study. The subjects had been diagnosed with cystic fibrosis in Hacettepe University Medical Faculty Department of Child Pulmonary Diseases by clinical findings consistent with cystic fibrosis and a sweat chloride value above 60 mEq/L on two measurements or cystic fibrosis-relevant mutations on both alleles of the CFTR gene, an age of ≥ 13, a forced expiratory volume in 1s (FEV1) of ≥ 35%, and the ability to perform physical activity. The criteria for inclusion were the following: clinically stable, no medical contraindications for exercise testing and participating in an exercise training program. Home medication (inhaled or oral antibiotics, bronchodilators, pancreatic enzyme supplements, and vitamins) was continued unchanged throughout the time of study. Exclusion criteria were non-cystic fibrosis related chronic disease and cystic fibrosis related conditions posing an increased risk to the patient when exercising. The scope and aim of the study were explained to the children participating in the study and their families and written informed consent was obtained. All procedures were in accordance with the current revision of the Helsinki Declaration [13].

Four patients were excluded from the study at the base line assessment due to a FEV1 below 35%. No patients were excluded for any other exclusion criteria.

All assessments were done by a single qualified physiotherapist and all the evaluation was done by a single pediatric pulmonologist. Exercise training and chest physiotherapy was done by another qualified physiotherapist.

The Schwachman scoring system [14] was used to determine the disease severity, clinical course and therefore the prognosis in cystic fibrosis cases while the Chrispin and Norman radiological scoring system was used to evaluate radiological changes [15].

All cases were evaluated before the physiotherapy program and after 6 weeks. The chest circumference was measured at three different points to evaluate the thoracic mobility.

The progressive multilevel treadmill test was done using modified Bruce, which is an incremental protocol, to determine the cardiovascular endurance of children with cystic [16]. The Enraf-Nonius (The Netherlands) treadmill was used for the test. The first two stages of the Modified Bruce Test are performed at a 1.7 mph and 0% grade and 1.7 mph and 5% grade, and the third stage corresponds to the first stage of the Standard Bruce Test protocol which continues as 1.7 mph 10% grade, 2.5 mph 12% grade, 3.4 mph 145 grade. Per stage takes 3 minutes.

The sit-up test was used to evaluate the dynamic endurance of abdominal muscles [17-19]. The standing long jump was used to test the power [17-19]. The sit and reach, trunk lateral flexion, trunk hyperextension, trunk rotation and forward bending tests were used to assess flexibility in patients with cystic fibrosis [17-20].

The subject was asked to run a 20 m distance as fast as possible in the 20 m shuttle run test. The running time was recorded with a chronometer [17,19].

The 10-step stair climbing test consisted of asking the subjects to climb the stairs without skipping any steps and using one foot for each step and descend without stopping and the duration was recorded. The height of the step was standardized as 15 cm.

Patients were trained three times a week for six weeks. At each visit they underwent the active cycle of breathing techniques and aerobic exercise training program on the treadmill. The exercise training was on the treadmill using 75-80% of the maximum heart rate for 30 min. The workload during the training was obtained by using the heart rate, blood pressure, electrocardiography (ECG) changes and clinical symptoms for each individual. The active cycle of breathing techniques was repeated three times for each pulmonary segment. The active cycle of breathing techniques and posture exercises were combined with breathing and taught to the families as a home program. There was no exacerbation during the study.

The data was expressed as mean ± standard deviation, The Wilcoxon test was used to compare the values before and after the physiotherapy program [21]. The significance level was accepted as p < 0.05.

## Results

The physical and clinical characteristics of the children included in the study are presented in table 1. Ten subjects were good, three mild and three were moderate according to their Shwachman scores as shown in table 1. The parameters related to the treadmill test such as blood pressure, heart rate, inclination and average speed is given in table 2.

**Table 1:** Characteristics of the patients

	Mean ± SD	Min-max
Age (years)	8.25 ± 2.77	5-13

Length (cm)	120.68 ± 15.15	107-153

Weight (kg)	22.17 ± 7.63	15-39

Shwachman Clinical Score	73.93 ± 12.40	47-90

Crispin and Norman Radiologic Score	10.06 ± 5.06	4-20

VC	89.37 ± 13.81	72-107

FEV1 (%)	87.15 ± 13.12	80-109

%FEF_25-75 _(%)	87.25 ± 14.89	61-112

VC- vital capacity, FEV1 (%)- Forced expiratory volume in one second, %FEF_25-75 _(%)- %25-75 of forced expiratory flow.

Datas are presented as mean ± standart deviation, median (min-max).

**Table 2:** Heart rate, blood pressure, speed and inclination values of the treadmill test.

	Before training (Mean ± SD)	After training (Mean ± SD)	P
Speed (km/hr)	6.41 ± 1.22	8.5 ± 0.89	0.030

Inclination (%)	15.50 ± 2.0	18.75 ± 2.05	0.020

Heart rate (beats/min)			

Initial	115.81 ± 14.66	111.68 ± 12.18	0.244

Final	182.43 ± 8.32	179.43 ± 12.15	0.461

Recovery 3. min	122.93 ± 12.79	111.18 ± 11.25	0.007

Recovery 5. min	120.12 ± 11.99	109.50 ± 13.10	0.008

Blood Pressure (mmHg)			

Initial	103.12 ± 9.46	90.31 ± 10.40	0.002

Final	132.50 ± 15.27	126.56 ± 7.23	0.063

Recovery 3. min	101.56 ± 10.60	92.50 ± 12.90	0.025

Recovery 5. min	96.25 ± 8.06	89.37 ± 11.23	0.026

Datas are presented as mean ± standart deviation, p < 0.05

There was a statistically significant increase after the treatment in chest circumference from subcostal, epigastric and axillary regions and the sit-up, standing long jump, right lateral flexion, left lateral flexion, right rotation, left rotation, trunk hyperextension, 20 m shuttle run, and 10-step stair climbing tests compared to the pre-treatment state as shown in tables 3 and 4 (p < 0.05). No statistically significant difference was obtained in sit and reach and forward bending test (p > 0.05).

**Table 3:** Chest circumference of the patients.

	Before training Mean ± SD	After training Mean ± SD	p
Axillar difference (cm)	3.68 ± 1.74	5.37 ± 2.39	0.003

Epigastric difference (cm)	3.25 ± 1.39	5.81 ± 2.66	0.001

Subcostal difference (cm)	2.84 ± 1.56	5.25 ± 2.14	0.001

Datas are presented as mean ± standart deviation, p < 0.05

**Table 4:** Physical fittness parameters

	Before training		After training		z	P
			
	Mean	SD	Mean	SD		
Sit-ups (times/min)	25.87	7.5	28.25	7.43	-2.274	0.023

Standing long jump (cm)	89.31	29.49	98.37	29.52	-2.986	0.003

Sit and reach (cm)	3.18	3.70	3.81	3.48	-1.349	0.177

Forward bending (cm)	1.5	6.05	3.37	4.78	-1.307	0.191

Right lateral flexion (cm)	11.25	4.62	13.87	4.95	-2.604	0.009

Left lateral flexion (cm)	11.31	4.15	14.25	4.46	-2.950	0.003

Right rotation (cm)	13.50	5.40	15.06	4.55	-2.284	0.022

Left rotation (cm)	13.37	5.56	15.50	4.03	-2.484	0.013

Trunk hyperextantion (cm)	14.62	6.94	17.75	7.83	-3.320	0.001

20 m shuttle run (sn)	5.81	0.98	5.00	1.03	-2.648	0.008

10 step stair climbing (sn)	7.12	1.08	5.50	0.73	-3.477	0.001

SD: Standard deviation, p < 0.05

## Discussion

It was found that active cycle of breathing techniques used together with exercise training in clinically stable young cystic fibrosis patients increases thoracic mobility and the physical fitness parameters such as muscle endurance, strength, flexibility and speed. This study was done in young children with cystic fibrosis in contrast the existing studies which were done in adolescents and adults.

Cystic fibrosis is a disorder that affects many systems and can cause various problems. Respiratory system involvement is expected in all patients and plays a significant role in determining the survival and quality of life in these patients. Pulmonary rehabilitation programs therefore play a large role besides medical treatment for treating children with cystic fibrosis. The general aims of pulmonary rehabilitation programs are to increase bronchial secretion clearance, exercise tolerance, and quality of life while decreasing the feeling of breathlessness. The control or decrease of these problems require developing a proper rehabilitation program using bronchial drainage, breathing techniques, easier coughing, respiration-improving postures and relaxation techniques [9,22-27].

Exercise is an essential treatment approach for cystic fibrosis patients because of psychosocial support and beneficial effects on the cardiovascular and pulmonary systems. Therefore the number of studies on cystic fibrosis has increased recently [28,29].

Tugay [30] has demonstrated in his study that children with cystic fibrosis scored lower values in sit-ups, sit and reach, forward bending, right and left trunk rotation and trunk hyperextension tests compared with their healthy peers. We concluded that these parameters can be improved with aerobic exercise training and respiratory exercises.

We found a 3 repeat/min increase in the push-up test where we evaluated muscle endurance. There are studies reporting increased muscle power and endurance with cardiovascular endurance training [31]. This result therefore supports our study. Exercise training on the treadmill is known to have a beneficial effect on increasing lower extremity and body muscle power [32]. We believe that the aerobic exercise program we provided in the study helped to develop muscle endurance. Sahlberg et al. [28], have found a decrease in quadriceps muscle strength and number of sit-ups after cardiovascular endurance training in their study on adult patients with cystic fibrosis. They have provided training to cystic fibrosis patients aged between 16-35, while the ages of the children in our study were between 5-13 years. It is thought that including children with cystic fibrosis in an aerobic exercise program in the early stage is important for developing muscle endurance.

We found that in young children with cystic fibrosis, aerobic exercise program combined with active cycle of breathing techniques increases strength. It is shown that increased cardiovascular endurance is effective in increasing the anaerobic strength of the individuals [33]. We feel that the increased strength of children with cystic fibrosis following the physiotherapy program is related to the aerobic training provided. Even the metabolism in cystic fibrosis is different from their healthy peers; all the children in our study were not severe and younger than the children recruited in other studies. The 20 m shuttle run and 10-step stair climbing tests evaluate both anaerobic power and speed. Exercise training in cystic fibrosis patients has been reported to have beneficial effects on the cardiorespiratory system [34,35]. It is not possible to consider aerobic and anaerobic strength independently as development in one area influences the other [33,36]. We saw that aerobic endurance training also increases anaerobic strength.

The aim of the active cycle of breathing techniques was to increase thoracic expansion. However, posture exercises combined with breathing techniques increased thoracic mobility and therefore body flexibility. Although the age ranges were different, there are two studies supporting our results that aerobic exercise training increases myofibril diameter and length [37,38]. A study showing an increase in flexibility with endurance training also supports our results [34]. We found improvements in all tests in children with cystic fibrosis except for the sit and reach and the forward bending tests. The absence of a difference in only these two flexibility tests may be due to the lack of stretching exercises dedicated to these muscles in the treatment program and the increased muscle tone especially in hamstring groups following the training on treadmill.

There is a study in the literature showing that postural reeducation has improved respiratory muscle strength, thoracic expansion and abdominal mobility [39]. Similarly we found that, active cycle of breathing techniques combined with aerobic training and posture exercises increased thoracic mobility and exercise tolerance in clinically stable cystic fibrosis patients.

One limitation of this study was the low number of children with cystic fibrosis included in the group and the lack of a control group. Future studies may reach comprehensive results using more detailed statistical analyses by increasing the number of children, control group and longitudinal follow up. Also learning effect can be eliminated by using cycle ergometer for the assessment and training on treadmill.

## Conclusions

It is thought that in addition to medical approaches to the systems affected, the active cycle of breathing techniques along with aerobic training helps to enhance the aerobic performance, thoracic mobility and improves physical fitness in children with cystic fibrosis.

## Competing interests

The authors declare that they have no competing interests.

## Authors' contributions

NT conceived and designed the study. BE was responsible for history taking, clinical examination and implementation of the chest physiotherapy and exercise training for children with cystic fibrosis. He analyzed the data, wrote the manuscript and took part in revision and submission. ID revised the manuscript for important intellectual content and took part in data analysis. UO helped in clinical examination, diagnose of children and selection of the children with cystic fibrosis as well as final revision of the manuscript. All authors read and approved the final manuscript.
